# Aluminum tolerance mechanisms in Kenyan maize germplasm are independent from the citrate transporter ZmMATE1

**DOI:** 10.1038/s41598-020-64107-z

**Published:** 2020-04-30

**Authors:** Thomas K. Matonyei, Beatriz A. Barros, Roberta G. N. Guimaraes, Evans O. Ouma, Reuben K. Cheprot, Leandro C. Apolinário, Dickson O. Ligeyo, Marcella B. R. Costa, Beatrice A. Were, Peter O. Kisinyo, Augustino O. Onkware, Roberto W. Noda, Samuel O. Gudu, Jurandir V. Magalhaes, Claudia T. Guimaraes

**Affiliations:** 1 University of Kabianga, Agroforestry and Rural Development Department, P.O. Box 2030-20200, Kericho, Kenya; 2 Embrapa Milho e Sorgo, Rodovia MG 424 km 65, Sete Lagoas, 35701-970 Brazil; 3 Universidade Federal de São João del Rei, Campus Sete Lagoas, Rodovia MG 424 km 47, Sete Lagoas, 35701-970 Brazil; 4 Rongo University, P.O. Box 103-40404, Rongo, Kenya; 5 University of Eldoret, P.O. Box 125-30100, Eldoret, Kenya; 6 Faculdade Ciências da Vida, Av. Prefeito Alberto Moura, 12632 Sete Lagoas, MG Brazil; 7 Kenya Agricultural Research Institute, P.O. Box 450-30200, Kitale, Kenya; 8 Universidade Federal de Minas Gerais, Departamento de Biologia Geral, Av. Antônio Carlos, 6627, Belo Horizonte, 31270-901 Brazil

**Keywords:** Plant genetics, Agricultural genetics, Genetic linkage study

## Abstract

Aluminum (Al) toxicity on acid soils adversely affects maize yields, which can be overcome by combining soil amendments with genetic tolerance. In maize, ZmMATE1 confers Al tolerance via Al-activated citrate release, whereby citrate forms non-toxic complexes with Al^3+^ in the rhizosphere. Here, we investigated Al tolerance mechanisms in maize germplasm originated from Kenya based on quantitative trait loci (QTL) mapping. Five QTLs and four epistatic interactions explained ~51% of the phenotypic variation for Al tolerance. The lack of Al tolerance QTL on chromosome 6 and the much lower expression of *ZmMATE1* in both Kenyan lines than in Cateto Al237, which donates the superior allele of *ZmMATE1*, strongly indicate that this gene does not play a significant role in Al tolerance in neither parent. In turn, maize homologs to genes previously implicated in Al tolerance in other species, *ZmNrat1*, *ZmMATE3*, *ZmWRKY* and *ZmART1*, co-localized with Al tolerance QTL and were more highly expressed in the parent that donate favorable QTL alleles. However, these candidate genes will require further studies for functional validation on maize Al tolerance. The existence of Al tolerance mechanisms independent from *ZmMATE1* suggests it is possible to develop highly Al tolerant cultivars by pyramiding complementary Al tolerance genes in maize.

## Introduction

Maize (*Zea mays* L.), the most produced cereal in the world, is largely used as animal feed, biofuel and is an important staple food in Africa and Latin America^1^. Maize occupies approximately 24% of all farmlands in Africa and accounts for 73% and 64% of the total food demand in Eastern-Southern and Western-Central Africa, respectively^2^. In Kenya, maize is consumed by approximately 96% of the population and is mainly produced on acidic soil regions where abiotic stresses contribute to very low maize yields (1.0–1.5 t/ha)^3,4^.

Maize yields are greatly reduced by Al toxicity on acid soils^5^, which cover approximately 50% of the global arable lands^6^, mainly in tropical and sub-tropical regions. Under pH below 5.5, the rhyzotoxic form of Al, Al^3+^, becomes prevalent in the soil solution, damaging plant root systems^7–9^. Hence, Al toxicity impairs soil exploitation, reducing water and nutrient uptake and, consequently, grain yield. The low soil pH problem can be ameliorated by lime application. However, liming is most effective in the arable soil layers^10^, whereas deeper soil correction is more difficult and costly. Moreover, soil amendments increase the production costs and are inaccessible for most small-hold farmers in developing countries^11^. Thus, a sustainable solution to enhance grain yields on acid soils can be achieved by associating soil fertility management practices with the adoption of Al tolerant cultivars.

Al tolerance in maize is a complex trait controlled by a few Quantitative Trait Loci (QTLs)^12–15^. The strongest maize Al tolerance QTL is located at bin 6.00 on chromosome 6, controlling 16 to 30% of the genetic variance in biparental populations derived from the highly Al-tolerant line, Cateto Al237^15,16^. Even though several genes have been identified controlling different mechanisms of Al tolerance in plants^17^, copy number variation of the Al-activated citrate transporter, *ZmMATE1*, was found to underlie the Al tolerance QTL on chromosome 6^16,18^. *ZmMATE1* expression is up-regulated by Al and three copies in tandem of the transporter gene were associated with enhanced *ZmMATE1* expression and Al tolerance^18^. The superior allele of *ZmMATE1* improving Al tolerance was donated by Cateto Al237 and was only found in two other lines, one from Brazil and another one from Bolivia^18^. Thus, the rare, favorable allele of *ZmMATE1* is mainly present in maize lines from South America^18^.

Maize landraces and lines from Kenya also showed high Al tolerance in nutrient solution^19,20^, reaching levels similar to Cateto Al237, the Brazilian source of *ZmMATE1*. However, Kenyan maize genotypes showed a lower *ZmMATE1* expression level at 6 hours after Al treatment than Cateto Al237^21^, suggesting the existence of different Al tolerance mechanisms in Kenyan germplasm. The present study aimed at identifying QTL and candidate genes associated with these mechanisms in an F_2:3_ population derived from the highly Al-tolerant Kenyan line, 203B-14, crossed with SCH3 (Al-sensitive).

## Results

### Variability of aluminum tolerance in the Kenyan maize population

Significant genotypic variation was detected for Al tolerance in F_2:3_ progeny derived from 203B-14 (Al tolerant) crossed with SCH3 (Al sensitive) based on relative net root growth (RNRG) (Supplementary Table S1). The heritability for Al tolerance based on family means was high (0.97), and the coefficient of experimental variation was low (8.82%) (Supplementary Table S1), indicating high quality of the phenotypic data.

**Figure 1:**
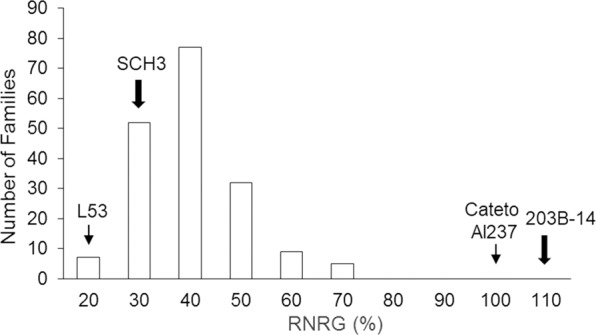
Distribution of aluminum tolerance based on relative net root growth (RNRG %) in Kenyan maize progeny. The population was composed by 180 F_2:3_ progeny derived from SCH3 (Al-sensitive) × 203B-14 (Al-tolerant). RNRG means of Kenyan parents are shown by thick arrows, whereas that of the Brazilian standards for Al sensitivity and Al tolerance, L53 and Cateto Al237, respectively, are depicted by thin arrows.

The RNRG population mean was 35.2% (Supplementary Table S1) with minimum and maximum values of 17.6 and 68.3%, respectively (Fig. 1). The RNRG mean of the Al-tolerant parent 203B-14, 107.7%, was similar to the Brazilian Al-tolerant maize line, Cateto Al237 (95.2%), but higher than the most Al-tolerant F_2:3_ progeny (Fig. 1). On the other hand, the Al-sensitive parent, SCH3, was less sensitive to Al than the most Al-sensitive F_2:3_ progeny and the Brazilian Al-sensitive line L53 (Fig. 1).

### Al tolerance QTLs

**Figure 2:**
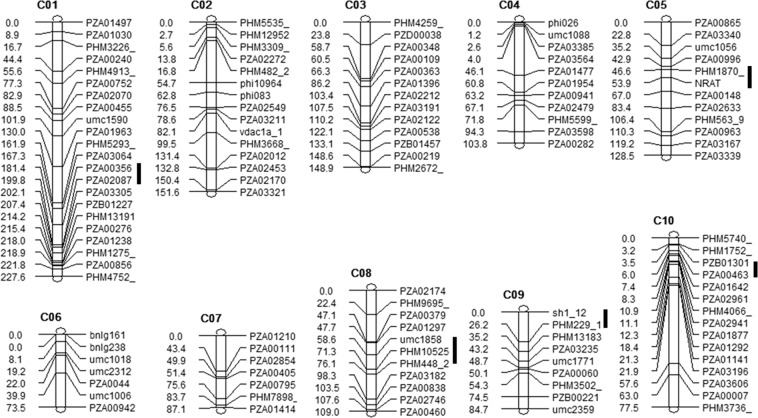
Aluminum tolerance QTLs in Kenyan maize population. The F_2:3_ 203B-14 × SCH3 linkage map was composed by 122 markers distributed along the 10 maize chromosomes. QTLs are depicted by black lines on the right hand side of the map, whereas genetic distances based on the Kosambi function (in centiMorgans) are shown on the left.

Out of the 198 markers genotyped in the population (183 SNPs, 14 SSRs and the ZmNrat1), 122 Mendelian markers (i.e. single locus segregation frequencies as expected for an F_2_ population) were used in the linkage map, covering 1,192.2 cM of the ten maize chromosomes (Fig. 2). Chromosome 1 had the highest number of markers (22) spanning 227.6 cM, whereas chromosome 7 had only seven markers along 87.1 cM. Four gaps ranging from 35 to 42 cM were present on chromosomes 2, 4, 7 and 10. The order of the markers flanking these gaps was confirmed by their physical positions according to MaizeGDB.

Five Al tolerance QTLs were mapped to chromosomes 1, 5, 8, 9 and 10, individually explaining up to 5.5% of the phenotypic variance for RNRG (Table 1). A model with QTL main effects and four epistatic interactions explained 50.94% of the phenotypic variance for Al tolerance. The Al-tolerant parent (A) donated alleles increasing Al tolerance for three QTLs (*qALT1.09*, *qALT5.03* and *qALT10.02*; Table 1 and Supplementary Fig. S1a), whereas the sensitive parent allele (B) increased Al tolerance for *qALT8.05* and *qALT9.01* (Table 1 and Supplementary Fig. S1b). For *qALT1.09*, *qALT8.05* and *qALT9.01*, heterozygous progeny had lower RNRG than the mean of both homozygous classes (Fig. S1a,b), which resulted in dominance effects with negative signs (Table 1). On the other hand, heterozygous progeny for *qALT5.03* and *qALT10.02* showed higher RNRG than the mean of homozygous progeny (Fig. S1a).

**Table 1:** Aluminum tolerance QTLs detected in an F_2:3_ population derived from 203B-14 x SCH3 based on multiple interval mapping.

QTL	Bin^a^	Marker^b^	Position^c^		LOD^d^	Type^e^	Effect^f^	R^2^ (%)^g^
			cM	Mb				
*qALT1.09*	1.09	PZA00356_8	219.7	263.6	3.91	A	3.79	5.5
					1.72	D	−3.43	2.7
*qALT5.03*	5.03	PZA001870_20	55.8	59.3	3.49	A	3.71	5.4
					0.61	D	2.15	1.2
*qALT8.05*	8.05	PHM10525_9	84.8	124.7	0.09	A	−0.64	0.5
					2.63	D	−4.07	4.0
*qALT9.01*	9.01	PHM229_15	27.0	30.0	2.35	A	−3.28	5.5
					0.96	D	−3.25	2.2
*qALT10.02*	10.02	PZB01301_5	3.3	9.7	2.76	A	2.85	4.8
					0.34	D	1.46	0.3
**Interactions**								
*qALT8.05* × *qALT1.09*					2.82	DD	8.77	4.8
*qALT8.05* × *qALT9.01*					2.68	DD	7.54	3.6
*qALT10.02* × *qALT1.09*					2.18	DA	−5.45	3.3
*qALT1.09* × *qALT9.01*					1.47	DA	7.53	7.0
**Total R**^**2**^ **(%)**								**50.94**

^a^Bin: segments of genetic maize map designated by the chromosome number followed by a two-digit decimal.

^b^The closest marker upstream to the QTL peak.

^c^Marker position based on the genetic map in centiMorgans (cM) and the B73 genome sequence version 4.0 in mega base pairs (Mb).

^d^Logarithm of odds score for the QTL effects. QTLs were significant based on the Bayesian Information Criterion.

^e^A: additive or D: dominance effects.

^f^Positive additive effect implies that the QTL allele derived from 203B-14 increases Al tolerance, whereas the negative sign indicates that SCH3 donates the QTL allele increasing Al tolerance. Positive dominance effect indicates that heterozygous progeny are more tolerant than the homozygous progeny and the negative dominance effect indicates that heterozygous progeny are less tolerant than the homozygous progeny.

^g^R^2^ is the proportion of phenotypic variance explained by each QTL, and total R^2^ is the phenotypic variance explained the full QTL model.

**Figure 3:**
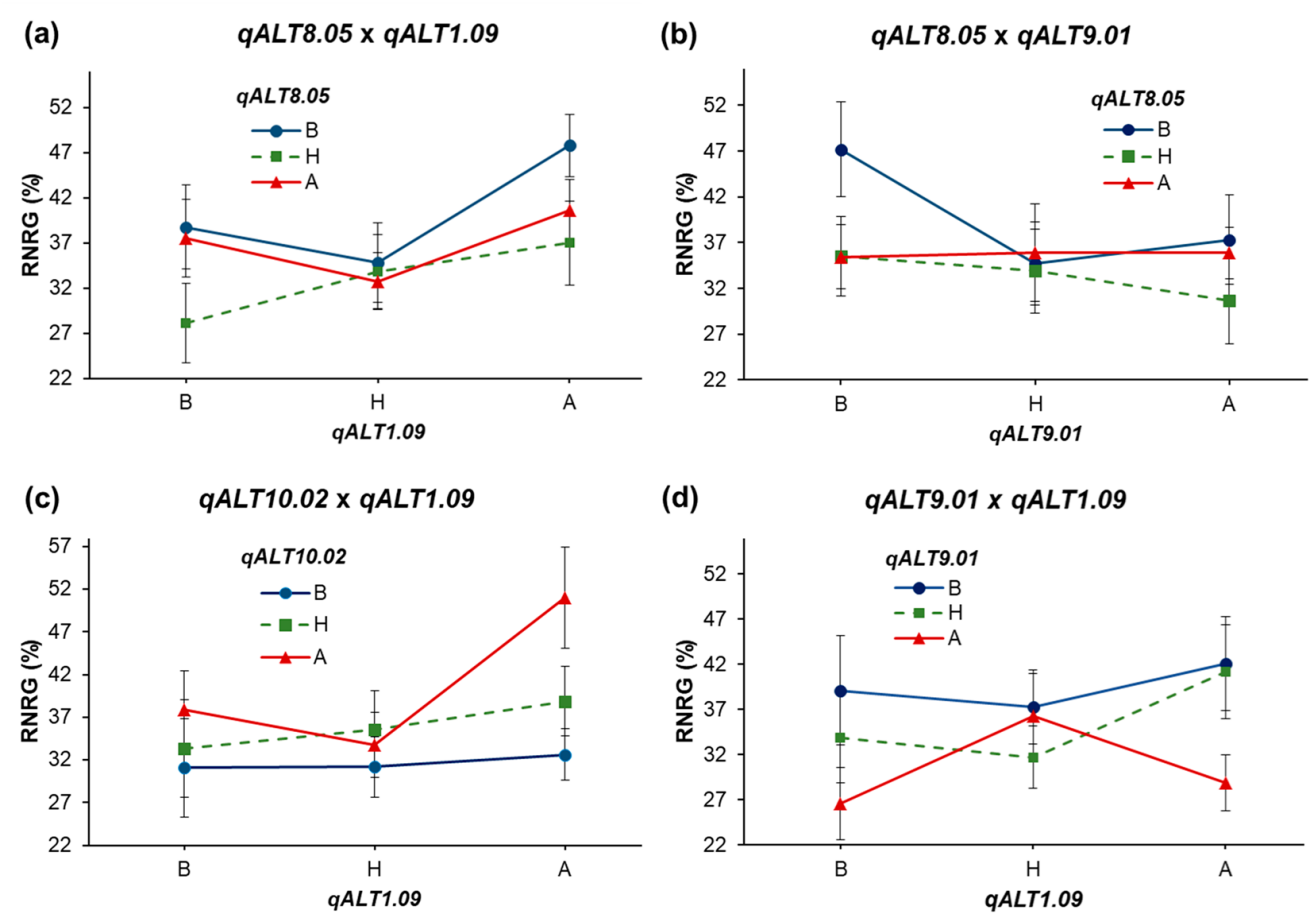
Al tolerance (RNRG means and standard deviations) for F_2:3_ progeny showing combinations of alleles at pairs of epistatic QTLs. Progeny homozygous for the allele donated by the Al-sensitive parent, SCH3, are shown in B (blue dot), whereas progeny homozygous for the Al-tolerant parent, 203B-14, are shown in A (red triangle). Means for heterozygous (H) progeny are also shown (green square). Interaction between QTLs **(a)** *qALT8.05* × *qALT1.09*, **(b)** *qALT8.05* × *qALT9.01*, **(c)** *qALT10.02* × *qALT1.09* and **(d)** *qALT9.01* × *qALT1.09*.

Epistatic interactions between QTLs explained from 3.3 to 7.0% of the phenotypic variance for RNRG (Table 1), which was similar to the individual QTL main effects. In both epistatic interactions involving *qALT8.05* (*qALT8.05* × *qALT1.09* and *qALT8.05* × *qALT9.01*), F_2:3_ progeny homozygous for the Al-sensitive (SCH3) allele at *qALT8.05* showed the highest RNRG mean (blue dots, Fig. 3a,b). For the interaction *qALT10.02* × *qALT1.09*, the most Al tolerant progeny were homozygous for alleles donated by the tolerant parent at both QTLs (red triangles and A, Fig. 3c). The interaction between *qALT9.01* × *qALT1.09* showed that most of the Al sensitive progeny carried the unfavorable allele at *qALT9.01* (Fig. 3d).

### Expression pattern of *ZmMATE1*

**Figure 4:**
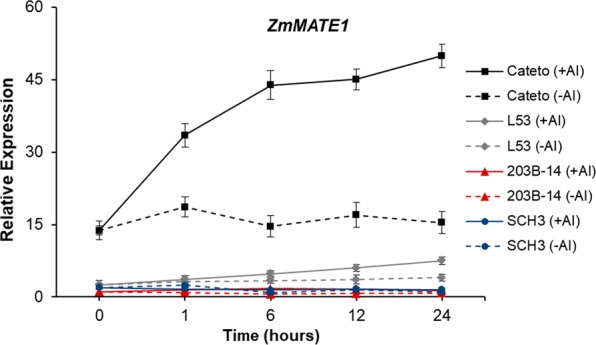
Expression profile of *ZmMATE1* in the parental maize lines from Kenya 203B-14 (Al-tolerant) and SCH3 (Al-sensitive) as well as in Cateto Al237 (Al-tolerant) and L53 (Al-sensitive). RNA was isolated from root apices (1 cm) sampled from seven seedlings for each biological replicate after 0, 1, 6, 12 and 24 hours of cultivation without (−Al) and with (+Al) {39} µM Al^3+^ activity in nutrient solution. 203B-14 cultivated without Al at time 0 was used as reference. Error bars depict the standard deviation of three biological replicates.

The lack of an Al tolerance QTL at bin 6.00 suggests that *ZmMATE1* does not play a role in Al tolerance in the 203B-14 x SCH3. Then, the time course expression of *ZmMATE1* in these parents was compared to the Al-tolerant Cateto Al237 and the Al-sensitive L53. The expression of *ZmMATE1* in the root tips of both Kenyan lines was much lower than in Cateto Al237, which donates the superior allele of *ZmMATE1*, and was even lower when compared to the Al-sensitive line from Brazil, L53 (Fig. 4). The time course expression of *ZmMATE1* in the Brazilian maize lines was in agreement with Maron *et al*.^16^.

### Candidate genes co-localized with Al tolerance QTLs

The number of predicted genes within each Al tolerance QTL ranged from 69 to 447 (Table 2), which were described in the Supplementary Table S2. We selected one to two candidates within each QTL interval based on similarity with other genes previously characterized for Al tolerance in other species. No previously characterized Al tolerance genes were found within the *qALT1.09* and *qALT9.01* intervals, suggesting that new candidate genes underlie these QTLs.

**Table 2:** Selected candidate genes within each Al tolerance QTL interval, including their physical positions in mega base pairs (Mb) and their putative homologs.

QTL	Chr	QTL Interval			Candidate Gene		
		Flanking Markers	Position (Mb)	Number of predicted genes	Gene ID	Position (Mb)	Homologs
*qALT1.09*	1	PZA00356_8	263.6	447			
		PZA02087_2	285.1				
*qALT5.03*	5	PHM1870_20	59.3	262	GRMZM2G065154	71.7	*ZmMATE1*
		ZmNrat	74.6		GRMZM2G168747	74.6	*OsNrat1*
*qALT8.05*	8	umc1858	111.2	401	GRMZM2G034421	118.5	*SbWRKY1*
		PHM448_23	133.8				
*qALT9.01*	9	sh1_12	11.3	407			
		PHM229_15	30.0				
*qALT10.02*	10	PZB01301_5	9.7	69	GRMZM2G068710	10.1	*OsART1*
		PZA00463_3	13.5				

**Figure 5:**
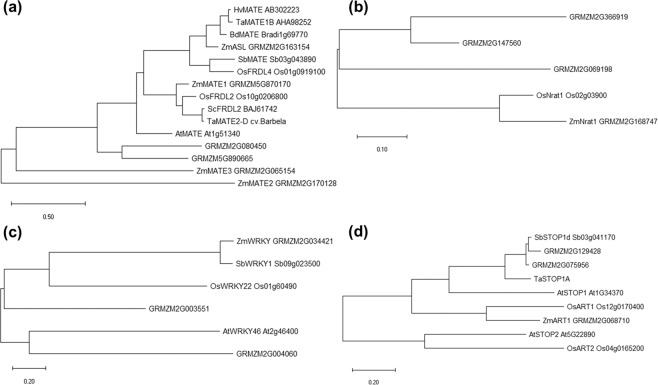
Phylogenetic trees of maize proteins predicted within the Al tolerance QTLs. Phylogenetic trees were based on maximum likelihood using the predicted amino acid sequences of maize candidate genes located within the Al tolerance QTLs, maize predicted gene with sequence identity higher than 21.8% and genes controlling Al tolerance in other plant species. (**a**) MATE transporters from rice, sorghum, barley, wheat, *Brachypodium*, *Arabidopsis* and maize. (**b**) Nrat1 aluminum transporters from rice and maize. (**c**) WRKY transcriptional regulators of Al tolerance genes from sorghum, rice, Arabidopsis and maize. (**d**) Transcriptional factor containing C2H2 motifs from sorghum, wheat, Arabidopsis, rice and maize.

The *qALT5.03* interval extended from 59.3 to 74.6 Mb on chromosome 5, and harbored two candidate genes, GRMZM2G065154 at 71.7 Mb and GRMZM2G168747 at 74.6 Mb (Table 2). GRMZM2G065154 encodes a MATE member (ZmMATE3) that shared 21.9% of sequence identity with ZmMATE1 (Supplementary Table S3), and clustered together with other citrate transporters associated with Al tolerance in several plant species (Fig. 5a). GRMZM2G168747 (ZmNrat1) had 81.8% sequence identity with OsNrat1 (Fig. 5b and Supplementary Table S3), an Al^3+^ transporter involved in rice Al tolerance^22^.

*qALT8.05* spanned a genomic region ranging from 111.2 to 133.8 Mb on chromosome 8, where GRMZM2G034421, which encodes a WRKY protein, was found at 118.5 Mb. GRMZM2G034421, designated henceforth as ZmWRKY, had 80.1% sequence identity with SbWRKY1 (Fig. 5c and Supplementary Table S3), a sorghum transcription factor that regulates *SbMATE* expression^23^.

GRMZM2G068710 is located at 10.1 Mb on chromosome 10, which is coincident with the *qALT10.02* interval (9.7–13.5 Mb). GRMZM2G068710 is 59.4% similar to OsART1 (Fig. 5d and Supplementary Table S3), a transcriptional regulator of Al tolerance genes in rice^24^, and is predicted to contain C2H2 zinc finger motif.

### Expression pattern of candidate genes in the parental lines

**Figure 6:**
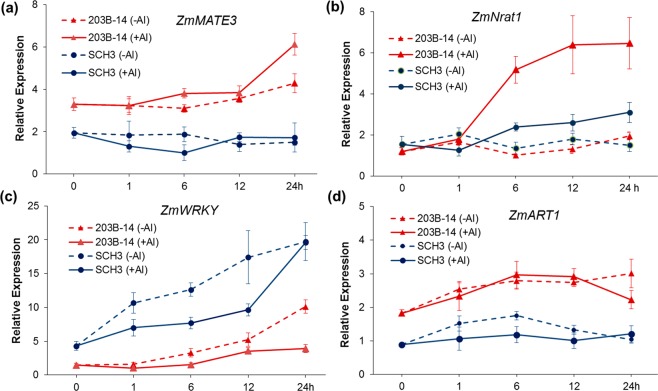
Expression profile of the candidate genes co-localized with Al tolerance QTLs. The contrasting parents are 203B-14 (Al-tolerant) and SCH3 (Al-sensitive) treated without (−Al) and with (+Al) {39} µM Al^3+^ activity in nutrient solution along 0, 1, 6, 12 and 24 hours (h). RNA was isolated from root apices (1 cm) sampled from seven seedlings for each biological replicate. Error bars depict the standard deviation of three biological replicates. **(a)** Relative expression of *ZmMATE3*, **(b)** *ZmNrat1*, **(c)** *ZmWRKY1*, and **(d)** *ZmART1*.

Temporal expression of candidate genes co-localized with Al tolerance QTLs was assessed in the root tips of the Al-tolerant and Al-sensitive parents, 203B-14 and SCH3, respectively. *ZmMATE3* was up-regulated in the Al-tolerant line after 24 hours of Al treatment, and was expressed at higher levels in the Al-tolerant line compared to the Al-sensitive parent (Fig. 6a). *ZmNrat1* expression was induced by Al in the root tip of the Al-tolerant line after 6 hours, reaching a plateau between 12 and 24 hours of Al exposure (Fig. 6b). *ZmWRKY* expression was higher in the Al-sensitive than in the Al-tolerant line, and was induced by Al in the Al-sensitive parent (Fig. 6c). *ZmART1* was more highly expressed in the Al-tolerant than in the Al-sensitive parent but was neither induced by Al nor differentially expressed in any time-point (Fig. 6d).

## Discussion

Several landraces collected at maize growing areas in Kenya showed high levels of Al tolerance in nutrient solution, including 203B, which was originated from an acid soil region in Muranga County^19^. The Al tolerant parent, 203B-14, was derived from selfing the landrace 203B. The existence of genetic variability for Al tolerance in landraces adapted to local agro-ecological regions can be used for molecular genetics studies and for breeding purposes. In this context, highly contrasting Kenyan maize lines for Al tolerance were selected and crossed to generate an F_2:3_ population, which was used for QTL mapping.

The QTLs and QTL interactions detected in this Kenyan population explained approximately 51% of the total variance for Al tolerance, which was lower but comparable to a QTL mapping study in a Brazilian population derived from Cateto Al237 × L53 (63%)^15^. Al tolerance in Cateto Al237 and 203B-14, parents of both the Brazilian and the Kenyan populations, respectively, was similar (~100% RNRG), which makes these lines highly Al tolerant^15,19–21,25^. The slight bias of derived F_2:3_ progeny towards Al sensitivity could be explained, at least in part, by the presence of three epistatic interactions between QTLs. High Al tolerance was observed in progeny that are double homozygous for favorable alleles at each pair of interacting QTLs (Fig. 3a–c). Taking two unlinked QTL loci whose alleles are assorted randomly, only 1/16 of the progeny are expected to be double homozygous for the favorable alleles. Hence, very few progeny should be highly Al tolerant, whereas the majority would express from medium to low Al tolerance.

No Al tolerance QTL was mapped on chromosome 6 (bin 6.00), which harbored the Al tolerance gene, *ZmMATE1*^16^, whose expression was much lower in 203B-14 and SCH3 than in Cateto Al237. Consistent with these results, both Kenyan parental lines showed lower *ZmMATE1* expression 6 hours after Al stress compared to Cateto Al237^21^. As Al-induced citrate transporter expression in the root tips leads to *ZmMATE1*-mediated Al tolerance in Cateto Al237^16,18^, low *ZmMATE1* expression in 203B-14 strongly supports the lack of this functional allele in 203B-14. Thus, the high Al tolerance in this Kenyan line is likely controlled to a large extent by mechanisms other than Al exclusion mediated by ZmMATE1. As other Al-tolerant maize genotypes from Kenya also showed low *ZmMATE1* expression^21,25^, it is possible that functional *ZmMATE1* alleles are not present in Kenyan maize germplasm.

We selected candidate genes co-localized with Al tolerance QTLs, which will be discussed in the light of QTL effects and gene expression profiles. The Al tolerance *qALT5.03* explained 5.4% of the Al tolerance phenotype and two candidate genes *ZmMATE3* (GRMZM2G065154) and *ZmNrat1* (GRMZM2G168747) are located within this genomic region. A positive correlation between citrate exudation and Al tolerance (r = 0.51, P < 0.05) in 12 Kenyan maize accessions has been previously reported^25^, suggesting that Al exclusion mediated by citrate release takes place in Kenyan maize germplasm. Phylogenetic studies of maize MATE-like proteins grouped ZmMATE3 with other MATE members previously characterized as citrate transporters in different plant species (Fig. 4b and Guimaraes *et al*.^15^). Different to the early induction reported for *ZmMATE1* in Cateto Al237^16^, *ZmMATE3* was induced by Al after 24 hours in 203B-14, which donated the *qALT5.03* allele improving Al tolerance. As ZmMATE1 was not functional in this population, ZmMATE3 could provide such Al tolerance mechanism to 203B-14.

ZmNrat1 is 83% similar to OsNrat1, a plasma membrane Al^3+^ transporter required for Al detoxification in rice^22^. A reduction of *OsNrat1* expression in rice resulted in shorter roots with higher Al concentration in the cell wall of root tips of the Al-sensitive parent compared to the Al-tolerant parent under Al stress^26^. In addition, *OsNrat1* was found to underlie the Al tolerance QTL on rice chromosome 2^22,26^. *ZmNrat1* (GRMZM2G168747), which was also co-localized with *qALT5.03*, was up-regulated after 6 hour of Al exposure in the root tip of 203B-14, which donates the favorable *qALT5.03* allele. It is thus plausible to hypothesize that the induction of *ZmNrat1* in the root tip of the 203B-14 could lead to Al tolerance based on a mechanism similar to that controlled by rice Nrat1, via Al transport reducing Al^3+^ concentration in the cell wall of apical cells^22^. Therefore, ZmNrat1 and ZmMATE3 could coordinate internal detoxification and exclusion mechanisms of Al tolerance, contributing to the high level of Al tolerance in 203B-14, conferred by *qALT5.03*.

The physical position of *qALT8.05* (111.1–133.8 Mb) coincides with that of marker umc1202 at 109.2 Mb on chromosome 8, and umc1202 was linked with an Al tolerance QTL detected based on root re-growth and hematoxylin staining^14^. This suggests that *qALT8.05* is conserved in different maize populations. In the 203B-14 × SCH3 population, *qALT8.05* harbors GRMZM2G034421 that encodes a group III WRKY protein. ZmWRKY has 80% sequence identity with SbWRKY1, and 20.6% and 17.1% with OsWRKY22 and AtWRKY46, respectively. SbWRKY1 activates *SbMATE* expression in sorghum^23^, OsWRKY22 activates the expression of *OsFRDL4* in rice^27^, whereas AtWRKY46 is a transcriptional repressor of *AtALMT* in Arabidopsis^28^, with all these genes being shown to control Al tolerance in the target species. Interestingly, ZmWRKY was induced by Al and showed higher expression in the Al-sensitive (SCH3) compared to the Al-tolerant parent. SCH3 donated the favorable allele of *qALT8.05* that interacted with both *qALT1.09* and *qALT9.01* alleles, improving the RNRG in the population (Fig. 3a,b). These data suggest that *ZmWRKY* would be a candidate gene underlying *qALT1.09*, encoding an activator of Al tolerance genes located within *qALT1.09* and *qALT9.01* regions.

The Al tolerance QTL, *qALT10.02* (8.7–13.5 Mb), was coincident with an Al tolerance QTL flanked by umc130 at 13.6 Mb, which was associated with an Al tolerance QTL in an F_2_ population derived from C100–6, another highly Al-tolerant Cateto line^12^. The predicted gene GRMZM2G068710, located within the *qALT10.02* interval, encodes a C2H2 zinc finger protein with 59.4% of sequence identity to OsART1, a transcription factor that regulates several Al tolerance genes in rice^24^. ZmART1 was phylogenetically closer to OsART1 and clustered with ART1/STOP1 transcription factors from Arabidopsis (AtSTOP1^29^), sorghum (SbSTOP1^30^) and wheat (TaSTOP1A^31^), which have been shown to control Al tolerance in these species. Although controlling the expression of several genes under Al stress, *OsART1* and *AtSTOP1* were not responsive to Al in rice^24^ and Arabidopsis^28^, similarly to *ZmART1* that was not differentially expressed by Al in both Kenyan maize lines. However, *ZmART1* presented higher expression in 203B-14, which donated the favorable allele of *qALT10.02*. Additionally, *qALT10.02* interacted with *qALT1.09*, with progeny double homozygous for the 203B-14 alleles at both QTL showing high Al tolerance. Thus, *qALT10.02* allele from 203B-14 could enhance Al tolerance by harboring *ZmART1*, which would activate transcriptionally other genes.

A remarkable difference of this Kenyan population was the relative high effect of epistatic interactions between QTLs, which has never been detected in other mapping study of maize Al tolerance^12–15^. The existence of different Al tolerance mechanisms in Kenyan maize germplasm, independent from *ZmMATE1*, brings the opportunity to develop superior maize cultivars by introducing exotic lines harboring functional *ZmMATE1* allele, such as Cateto Al237. Al-tolerant cultivars should benefit maize production on acidic soil regions worldwide.

## Material and Methods

### Plant material

The plant material consisted of 180 F_2:3_ progeny derived from a cross between Kenyan maize inbred lines previously characterized as extremely tolerant (203B-14) and sensitive (SCH3) to Al^20^. Additionally, the Brazilian standard lines for Al tolerance (Cateto Al237) and sensitivity (L53) were used as checks in hydroponics.

### DNA extraction and markers genotyping

DNA was isolated from young leaves of the parental lines and F_2_ plants using a modified CTAB method as described by Saghai-Maroof *et al*.^32^. Genotyping of single nucleotide polymorphisms (SNPs) was performed using the Kompetitive Allele-Specific PCR (KASP^TM^) assays by LGC Genomics (www.lgcgenomics.com). The parental lines were screened with 1,250 random SNPs for polymorphism detection and F_2_ individuals were genotyped with 183 SNP markers.

Additionally, 14 fluorescently labeled SSR (Simple Sequence Repeat) markers were genotyped in the population. PCR reactions were performed using 50 ng of DNA, 1X PCR Buffer, 2.5 mM MgCl2, 166 µM of each dNTP, 0.2 μM of each primer and 0.5 U Taq DNA polymerase (Invitrogen, Thermo Fisher Scientific Inc.) in a final volume of 15 µL. Amplification cycles consisted of 95 °C for 2 minutes, eight cycles of 94 °C for 20 seconds, 60 °C (−1 °C/cycle) for 1 minute and 72 °C for 1 minute, followed by 35 cycles of 94 °C for 20 seconds, 53 °C for 1 minute and 72 °C for 1 minute, and a final extension of 72 °C for 5 minutes. PCR reactions were diluted in water (1:10), and 2 µL of each reaction were mixed with 0.3 µL of GeneTAB500 (Gene ID, São Carlos, Brazil) and 9.7 µL of 1% Tween 20. This mix was denatured at 94 °C for 5 minutes and loaded in a MegaBace 1000 DNA Analysis System (Amersham Biosciences, Thermo Fisher Scientific Inc.) with injection of 3 kV for 45 seconds and run at 10 kV for 75 minutes. The amplified fragments were analyzed using the software Fragment Profile 1.2 (Amersham Biosciences, Thermo Fisher Scientific Inc.). Sequence information and physical position of the SNPs and SSRs are available at Maize Genetics and Genomics Database (www.maizegdb.org/data_center/locus).

The PCR reaction to map *ZmNrat1* was performed with 30 ng DNA, 1X PCR Buffer, 2 mM MgCl_2_, 125 µM of each dNTP; 0,5 µM of each primer F: 5′CGCGGAAACAGGAACCAAACCAAAA3′ and R: 5′CGGGTCTCTGCGTACCCCGA3′, 5% DMSO and 1U Taq DNA polymerase (Invitrogen, Thermo Fisher Scientific Inc.) in a final volume of 20 µL. Amplification cycles consisted of 95 °C for 2 minutes, 30 cycles of 94 °C for 30 seconds, 65 °C for 30 seconds and 72 °C for 1.5 minute, and a final extension of 72 °C for 5 minutes. The PCR product was cleaved with *Hinf*I and the fragments were visualized in agarose gel 1.5% (w/v) stained with GelRed Nucleic Acid Stain (Biotium, Fremont, CA).

### Evaluation of aluminum tolerance in nutrient solution

Al tolerance was assessed in a growth chamber under nutrient solution according to Guimaraes *et al*.^15^. Briefly, four-day old seedlings were transferred to polyethylene cups organized into containers filled with nutrient solution^33^ at pH 4.0 under continuous aeration. After 24 h of acclimatization, the initial root length (IRL) was measured and the seedlings were cultivated with and without {39} µM of Al^3+^ activity supplied as AlK(SO_4_)_2_.12H_2_O (brackets denote free Al^3+^ activity estimated with GEOCHEM-EZ software^34^ that corresponds to 222 µM of Al concentration). The final root length (FRL) of each seedling was measured five days after the treatments and net root growth (NRG) was calculated as FRL – IRL under Al treatment (NRG_+Al_) and control conditions, without Al (NRG_−Al_). The phenotypic index used to evaluate Al tolerance was Relative Net Root Growth (RNRG) calculated as NRG_+Al_/NRG_−Al_(x100).

The F_2:3_ progeny and the parents were evaluated in six experiments carried out in a completely randomized design with three replicates and two common checks (Cateto Al237 and L53). Analysis of variance was performed with RNRG data using PROC GLM of SAS software version 6.1.7601. Broad sense heritability (*H*^2^) was estimated based on family means.

### Linkage analysis and QTL mapping

Marker loci were tested for goodness-of-fit to the expected single locus segregation ratio in an F_2_ population (1:2:1) using the chi-square test (P < 0.05). The linkage map was constructed using the MapMaker/EXP 3.0^35^, with a minimum LOD of 3.0 and a maximum recombination frequency of 0.4. The Kosambi mapping function^36^ was used to convert recombination frequencies into map distances in centiMorgans (cM).

QTL mapping was performed using multiple interval mapping (MIM)^37^ implemented in QTL Cartographer version 2.5 for Windows^38^. The final model was selected using forward selection based on the Bayesian Information Criterion (BIC) with the penalty function *c(n)* = *log(n)*, in which *n* = 180. The QTL position was defined based on the closest marker to the QTL maximum LOD value, and those markers were used to calculate the RNRG mean of genotypic classes for each QTL and for combinations of epistatic QTLs. Confidence intervals were established using the LOD-1 criterion^39^. The QTLs (*q*) were named using the acronym of Al tolerance (*ALT*) followed by their genetic position in chromosomal bins.

### Searching for candidate genes within the Al tolerance QTLs

Genes previously associated with Al tolerance in other species were searched within the confidence intervals of Al tolerance QTLs based on sequence similarity using Phytozome (phytozome.jgi.doe.gov) considering the B73 genome sequence version 4.0.

### Phylogenetic analysis of candidate genes

Protein sequences encoded by the candidate genes co-localized with Al tolerance QTLs and other similar sequences from maize were aligned with their respective homologs controlling Al tolerance in other plants using the M-COFFEE package available at T-COFFEE (tcoffee.crg.cat). Percent of identity was based on Clustal Omega (www.ebi.ac.uk/Tools/msa/clustalo/) and phylogenetic trees were constructed based on maximum likelihood using the software Mega v 10.0.5^40^.

### Expression analysis of candidate genes

The expression profiles of the candidate genes co-localized with Al tolerance QTLs were evaluated by quantitative real-time PCR (RT-qPCR) using the ABI Prism 7500 Fast System (Applied Biosystems, Thermo Fisher Scientific, Inc.). Maize seedlings were grown in hydroponics as described in the section *Evaluation of aluminum tolerance in nutrient solution* with seven seedlings representing each sample. The first centimeter of the root tips were collected after 0, 1, 6, 12 and 24 hours of treatment with and without {39} μM of Al^3+^ activity in the contrasting parents 203B-14 and SCH3. Total RNA was extracted using the RNeasy Plant Mini Kit (Qiagen, Germantown, MD) and the first-strand cDNA was synthesized using the High Capacity cDNA Reverse Transcription kit (Applied Biosystems, Thermo Fisher Scientific, Inc.) according to the manufacturer’s instructions. Transcripts were quantified using cDNA (5 ηg for target genes and 0.005 ηg for the endogenous control 18S rRNA), 2.5 μM of each primer and Fast SYBR Green Master Mix 1×(Applied Biosystems, Thermo Fisher Scientific, Inc.) in a final volume of 10 μL. Primers for each target gene were designed using the Primer-Blast tool (www.ncbi.nlm.nih.gov/tools/primer-blast/) (Supplementary Table S4). Calculation of relative gene expressions were performed using 2^−ΔΔCt^ method^41^, with three biological and three technical replicates for each biological sample.

## Supplementary information

Supplementary information.

Supplementary information2.

## Data Availability

The dataset for linkage and QTL mapping is included in this published article.
