# Supplementary material for: Aluminum tolerance mechanisms in Kenyan maize germplasm are independent from the citrate transporter ZmMATE1
Source: Sci Rep. 2020 Apr 30;10:7320. doi: 10.1038/s41598-020-64107-z (PMC7193623; doi:10.1038/s41598-020-64107-z)
Supplement: Supplementary file 1 — Supplementary information. [file 41598_2020_64107_MOESM1_ESM.pdf]

Aluminum tolerance mechanisms in Kenyan maize germplasm are independent from the citrate transporter ZmMATE1

Thomas K. Matonyei[1], Beatriz A. Barros[2], Roberta G.N. Guimaraes[3], Evans O. Ouma[4], Reuben K. Cheprot[5], Leandro C. Apolinário[6], Dickson O. Ligeyo[7], Marcella B. R. Costa[8], Beatrice A. Were[5], Peter O. Kisinyo[4], Augustino O. Onkware[4], Roberto W. Noda[2], Samuel O. Gudu[4], Jurandir V. Magalhaes[2], and Claudia T. Guimaraes[2]*

[1] University of Kabianga, Agroforestry and Rural Development Department, Kericho, P.O. Box 2030-20200, Kenya

[2] Embrapa Milho e Sorgo, Rodovia MG 424 km 65, Sete Lagoas, 35701-970, Brazil

[3] Universidade Federal de São João del Rei, Campus Sete Lagoas, Rodovia MG 424 km 47, Sete Lagoas, 35701-970, Brazil. Current address: Agroceres, Sorriso, MT, Brazil

[4] Rongo University, P.O. Box 103-40404, Rongo, Kenya

[5] University of Eldoret, P.O. Box 125-30100, Eldoret, Kenya

[6] Faculdade Ciências da Vida, Av. Prefeito Alberto Moura, 12632, Sete Lagoas, MG, Brazil

[7] Kenya Agricultural Research Institute, P.O. Box 450-30200, Kitale, Kenya

[8] Universidade Federal de Minas Gerais, Departamento de Biologia Geral, Av. Antônio Carlos, 6627, Belo Horizonte, 31270-901, Brazil

* Corresponding author: claudia.guimaraes@embrapa.br

Supplementary material

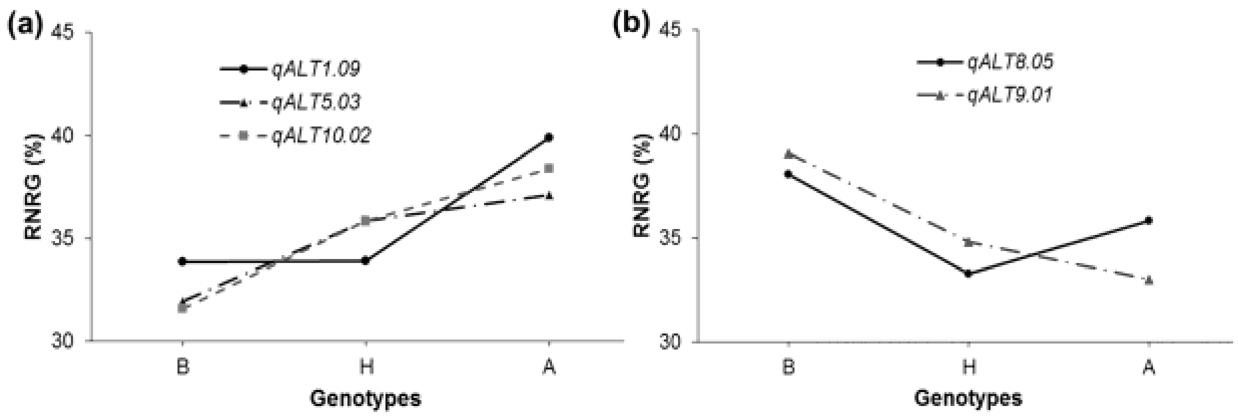

Supplementary Figure S1. Relative net root growth (RNRG %) mean for $F_{2:3}$ progeny showing genotypic classes of the Al tolerance QTLs alleles. Progeny homozygous for the allele donated by the Al-sensitive parent, SCH3, are shown in B, whereas progeny homozygous B for the Al-tolerant parent, 203B-14, are shown in A. Means for heterozygous are shown in H. **(a)** QTLs with favorable allele derived from 203B-14. **(b)** QTLs with favorable allele derived from SCH3.

Supplementary Table S1. Variance analysis for relative net root growth in the $F_{2:3}$ families derived from the cross 203B-14 x SCH3.

**Source of Variation**	**D.F.[a]**	**M.S.[b]**	**F[c]**
Experiments	5	1650.29	157.32**
Replicates/Experiments	12	26.86	2.56*
Checks x Experiments	5	103.66	9.88**
Families adjusted	183	715.48	68.11**
Checks	1	50912.11	4853.39**
Families	176	357.79	34.11**
Checks x Families	6	2841.73	270.90**
Residual	376	10.49	
Families Mean		35.21	
Coefficient of genetic variation (CVg%)		20.24	
Coefficient of experimental variation (CVe%)		8.82	
Broad sense heritability (H^2)		0.97	

[a] D.F. degrees of freedom
[b] M.S. mean square
[c] *, ** Significant at $p \leq 0.05$ and 0.01, respectively.

Supplementary Table S3. Maize predicted proteins similar to the Al tolerance genes previously characterized in plants (*ZmMATE1*, *OsNrat1*, *SbWRKY1* and *OsART1*). Percent of identity is based protein sequence alignment and genomic positons are in base pairs (bp).

Al Tolerance Gene	Gene ID[a]	Identity (%)	Chromosome	Position (bp)
ZmMATE1 (GRMZM5G870170)	GRMZM2G163154 (ZmASL)	56.4	1	27,099,590
	GRMZM5G890665	43.3	2	153,549,513
	GRMZM2G080450	40.3	3	57,084,587
	GRMZM2G065154 (ZmMATE3)	21.9	5	71,724,077
	GRMZM2G170128 (ZmMATE2)	21.8	5	20,622,821
OsNrat1 (Os02g03900)	GRMZM2G168747 (ZmNrat1)	81.8	5	74,616,918
	GRMZM2G147560	60.3	7	26,963,762
	GRMZM2G366919	52.6	2	170,851,138
	GRMZM2G069198	49.5	5	43,146,138
SbWRKY1 (Sb09g023500)	GRMZM2G034421 (ZmWRKY)	80.1	8	118,496
	GRMZM2G003551	30.3	9	18,108,182
	GRMZM2G004060	25.4	10	12,108,516
OsART1 (Os12g0170400)	GRMZM2G068710 (ZmART1)	59.4	10	10,146
	GRMZM2G075956	48.6	8	165,627
	GRMZM2G129428	46.8	3	174,504

[a] Gene names in parenthesis were given by Krill *et al*. (2010) for *ZmASL*, Maron *et al*. 2010) for *ZmMATE2* and the others genes are described in this current work.

References

Krill, A. M., Kirst, M., Kochian, L. V., Buckler, E. S. & Hoekenga, O. A. Association and linkage analysis of aluminum tolerance genes in maize. *Plos One*, e9958 (2010).

Maron, L. G. et al. Two functionally distinct members of the MATE (multi-drug and toxic compound extrusion) family of transporters potentially underlie two major aluminum tolerance QTLs in maize. *Plant J*. **61**, 728-40 (2010).

Supplementary Table S4. Sequence of the primers used for the expression analysis using quantitative RT-PCR of each candidate gene mapped within the Al tolerance QTLs.

Gene ID	Name	Primer Sequence 5´-3´
GRMZM2G065154	*ZmMATE3*	F - ATGACGTCCAAGGTGGCATT<br>R- TGATGGGCTGCAAGCGTTAT
GRMZM2G168747	*ZmNrat*	F - CGCGCTTCTGATCCAAACA<br>R - GCGAGATGCTTGCCTGTCTT
GRMZM2G034421	*ZmWRKY*	F - AGCTCATCCCGGAGCCTAA<br>R - GGAGAGCGACGACTCCATAG
GRMZM2G068710	*ZmART1*	F - CCCGTCACTGAAGAACTCGT<br>R - TCGCATTAGGCACTAGGCTC
18S rRNA		F - CGTCCTAGTCTCAACCATAAACG<br>R - CCCCGGAACCCAAAGACT
